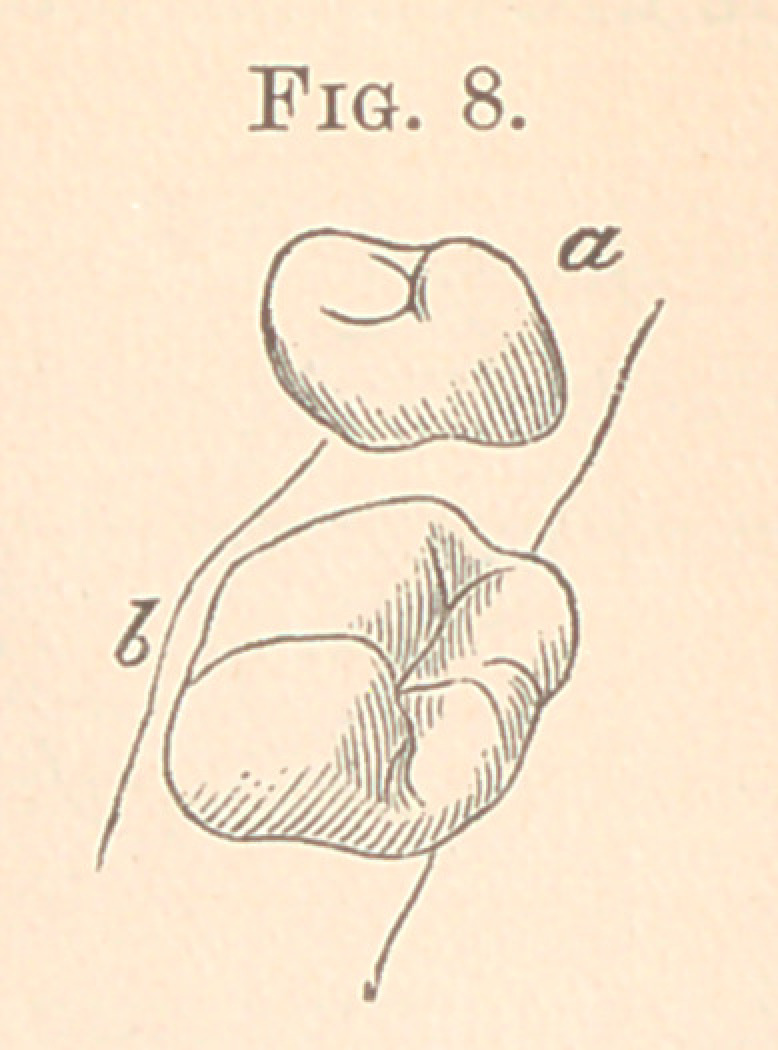# New Method of Clasped Plates *Versus* Movable or Unmovable Bridge-Work

**Published:** 1893-02

**Authors:** W. G. A. Bonwill


### NEW METHOD OF CLASPED PLATES *VERSUS* MOVABLE OR UNMOVABLE BRIDGE-WORK.[1]

BY W. G. A. BONWILL.

TIME, with experience, levels all things. How one's practice will be changed if truth be uppermost!

I am surprised at the many summersaults I have made in nearly forty years. Because the Fathers said "*Don't*," I did as they bade me, and should yet, perhaps, if I had not been made of the material of which revolutionists come.

At one time I would not put in an upper plate without a suction, and never a lower one with a clasp.

For eight years gold and tin were used, but never amalgam.

---

[1] Read before the Odontological Society of Pennsylvania, June 7, 1890.

The uses of contour, at first, I did not see. Its beauties were more apparent when kept out of sight. Also artificial dentures and flat grinding-surfaces. All through my early practice I was afraid of offending the Fathers, and it caused me much sorrow and heart-ache and kept me in poverty and made me work harder than a galley-slave.

But live as long as you may, there are to be found many young as well as old persons who say "Stop" to every new advance. Do not mind them, but strike out boldly for yourselves and humanity.

In one thing I never have changed from the beginning of my career, and that is, never to sacrifice a tooth without a struggle for its existence. A human tooth has always been treasured as a physician would the living body, and with the experience gained and ingenuity in overcoming difficulties I seldom, to-day, extract a tooth.

In the effort to make all plates with suction I had an experience in the loss of a superior left lateral from bad dentistry during my first year of practice, giving me an opportunity to know personally why it could not be used in mastication. I tried in vain. On one occasion, instead of removing it as had been my custom at night, I kept it in, and had finished breakfast the next day before I discovered the fact. There had been accomplished with the plate that which I had never been able to do,—keep it in my mouth while eating. This was suggestive, and I applied it to others, and avoided clasping the teeth. In using only suction-plates I allowed many mouths, especially in the lower jaw, to go toothless, and yet, finally, a summersault was turned, and clasps are now used as a *sine qua non.* Adversity came still further to my aid, and it compelled me to use clasps and avoid bridging. The loss of a first inferior molar from too much cutting of bone material to gratify a great contourist, when a flat surface filling was indicated, and the "too much use of the electro-magnetic mallet" to gratify my vanity, destroyed it. I hammered the life out of it, and finally, as no one could relieve me, it was extracted. There was some immediate recompense, for it gave me an opportunity to try my own discovery,—"rapid breathing,"—and this proved an entire success. This loss was a most fortunate one for my patients, as it led me to adopt for them a plan which but for this I should never have thought of.

Up to this time I had persisted in refusing to use a clasp upon any tooth, and hundreds of cases in the lower jaw, and many in the upper, were allowed to go toothless because I could not put in artificial teeth except by mutilation and permanent bridging, which I have never made in a single case.

All practitioners who were consulted in this case said nothing but bridging would answer. Necessity compelled a violation of a supposed law, and the result has been that in ten years I have been doing this class of work, no vacancy in the mouth having gone unchallenged. During all this time I had seen many cases of clasping good teeth, and the reason of decay and wear where the band was placed was apparent.

*First.* Any one who has any idea of pure mechanics in dentistry must admit that artificial dentures are placed in without any method, reflection, or planning,—certainly not as a mechanical or civil engineer would do in advance on paper,—and with no system to reach the greatest strength of plate and artistic appearance. It is astounding how persons ever get used to sets of teeth where no articulation has been given. True articulation is a sealed book to the majority.

*Secondly.* Bands are allowed to go too far up and under the gum-border. They are never wide enough. They have either too much spring or not enough for each individual case.

*Thirdly.* The clasp is allowed to move constantly up and down on the tooth until the tooth is worn very materially.

*Fourthly.* The clasp is soldered to the plate always in one spot, —on the anterior or distal surfaces of the tooth, just where most spring of clasp is needed.

*Fifthly.* The clasp has always been soldered on to the plate by fitting both to the plaster cast and soldering them immediately from that.

*Sixthly.* This plan of soldering never allows the plate to fit as it should, since the clasp draws the plate away from the tooth, and the tooth is forced out of its place and is never easy.

*Seventhly.* The value of the clasp is lost in not comprehending the exact relation it should bear to the plate.

*Eighthly.* While the clasp and plate may fit well, it is always a failure if the proper articulation is not in keeping with the opposing teeth. It causes all the strain to come upon the clasped teeth, when the plate should rest easily on the gum, and the clasp be firm yet not binding on the tooth.

*Ninthly. The clasp made to fit too closely to every part of the surface of the tooth enveloped results in decay, from the fine capillary surfaces made by the too near contact of clasp and tooth.*

*Tenthly.* From the imperfect soldering of plate and clasps the bands have to be made to closely hug the surface; and, from the narrow necks of most teeth capable of being clasped, the plate is pushed too hard up against the gums, and to make the plate remain in the mouth the clasps are required to be stiffer and are never quite comfortable. There is not only wear, but caries is produced from the driving of the gum away from the cervix.

To add to this experience of clasping, there have been many failures from permanent and removable bridges. To grind off the entire enamel from any sound or partially sound tooth or teeth, to place over it a gold cap for one or many teeth, is the most unjustifiable of vandalisms. When I was told that the second bicuspid and second molar of the inferior jaw must be shaped to place over them caps of gold to insert a first molar tooth, I rebelled at once, as it appeared a sacrilege and a disgrace to our art.

Out of all the cases of bridging that have come to my notice, not one has been perfectly articulated. The surfaces of the bicuspids and molars have been ground flat, and, where cusps were placed on, when the jaw made a lateral movement, there was not a buccal cusp touched. The up-and-down movement alone was of any value. The cusps of gold were unsightly, and not the least art was manifest in the arrangement or in the selection of the teeth. The cement placed between the caps of gold and the tooth never fills up the space, being pushed out or away. Especially is this true of those cases where a part of the face of the natural tooth is shown from the cutting away of the gold cap.

The cement is put in so thin that it is sure to wash, or be dissolved by the powerful capillary force exerted by the oral fluids.

To add to this misery, the cervical border is seldom free from constant irritation. It is only necessary to allude to the stench arising from the accumulations upon the surface of the gold, like barnacles on a ship's bottom.

A bridge can not be kept any cleaner than a plate that is removable, and I never saw one of the latter that did not have to be polished out of the mouth with the same care as polishing silverware.

The dentist who will learn to place in partial and full upper or lower sets as they should and can be, and give no trouble to the wearer, will never resort to bridging except in very favorable cases, and not then by ever mutilating a good tooth-crown for a gold cap.

When a few teeth in the mouth are left without crowns, place on artificial crowns that can be clasped to hold the plate or plates in position without any fear of falling out. It is astonishing how firmly one tooth, with a properly-fitting clasp, will hold a full set, upper or lower. I have repeatedly utilized an old root or roots with a porcelain crown, and, in time, should it be lost, the patient has become so accustomed to the plate that it is not missed.

Above all else, I assert, knowingly, that could the art of articulation have been carried out as I have demonstrated so often, bridging would never have come into such general use.

The objection to the system of bridging is that but few of even the best dentists are capable of performing the high class of work necessary to make it successful. It has been used in practice sufficiently long to show that there never was a more signal failure in any line of work, not even copper amalgam.

I have placed on many cases of bridging by the nut and bolt, a process by which the parts could be unscrewed and removed and repaired, and then the nut replaced, and be as tight as a piece of engineering.

When I advocated the cutting of the approximal surfaces of teeth to arrest or anticipate decay, a howl went up all over the land. Now that it is "the thing" to bridge, the same men that abused me for doing what I knew was correct have no conscience in regard to mutilating the enamel of any tooth to which they wish to attach a permanent band. Their gold caps glare in the light, and make vulgarity more pitiable and the dentist more contemptible. Teeth can be clasped, no matter how much they may be out of line or at an angle with the plate, and it will be seen that they will be of far more use and more artistic.

It is not necessary to fill the whole arch and palate with a plate, where a few teeth remain. A narrow, heavy plate, unyielding in character, will stay up just as well when confined alone to the alveolar border. Or where one, two, or three teeth in either jaw must be replaced, they will need but one full clasp and a very small plate to act as a saddle.

When the idea is once grasped of how a clasp should be fitted to a tooth without mutilating it, and how the clasp should be soldered to the plate, then dentists will see a new era dawning upon them.

Be it understood that I am not in opposition to all bridging, but only such as is done by those not familiar with mechanical work. It is then the human teeth have to pay the penalty for ignorance and false ideas of art. There is no occasion to ever deface a tooth, save to sometimes remove sharp angles to allow the clasp to be firm, which does no harm if the surface is polished again.

What can be done to obviate all this?

First, how should a clasp be fitted to the natural crown of a tooth to prevent future caries, and also prevent wear, and of what material and how heavy or light the metal, and how wide and at how many points upon the crown's surface should it touch to insure its steadfastness or security?

The thickness of metal is dependent upon the length of clasp, the width of same, and whether one or more clasps will be used to sustain the plate, or where there has to be very much spring to the clasp in passing over a crown that is very much out of perpendicular.

The metal should be of platinized gold only, without any lining of pure or twenty-two-karat gold soldered on it next to the crown. The metal should be loosely fitted to the crown on the plaster cast and afterwards fitted in the mouth directly upon the tooth and made to touch in at least four places. It should not be struck up to fit accurately every inequality of the surface, nor should pure gold first be fitted to the tooth by burnishing it on and then soldering that to the platinized gold.

If a clasp fits minutely all the surface of the crown, it makes of the minute space between the crown and clasp a capillary surface, and keeps the mucous secretions, as well as the fine food, forever in contact and with no space for circulation of the saliva. Whereas, if the band touches but a few places on the tooth-crown, it will rest just as firmly if it has been well fitted in the mouth and allowed to take its own position when tried upon the crown.

Capillary power made by surfaces very closely approximated is the surest means of producing caries. Where a space is left, the points that do touch are in absolute contact, and, aside from a slight wear on the tooth, the surface cannot decay as when there is an actual and close fitting. If made of fine soft gold, there would always be danger.

A clasp is not needed to grasp the crown very closely. The width of clasp should be as great as can be made, and to steady the plate without grasping it firmly. This will be a new idea to many.

Next to the clasp in importance is to know where it should be soldered to the plate, and on which side of the crown to allow it to go on and off, where the crown is very much out of perpendicular.

In this lies the principle part of the plan, and upon it depends entirely, or greatly, the success of the operation. The plate may fit perfectly, and also the clasp, but all is vain unless the point is known where to unite the band and plate.

This cannot be done unless a plaster impression is taken of both the clasp and the plate in the mouth, so that the exact relation is obtained. The impression of plaster is now run with plaster and sand and the case soldered. To make the whole thing a perfect development the little gold angular tip must be soldered either to the clasp or the plate to keep the clasp from moving up and down.

It should be made of very heavy platinized gold and fitted to the top of the crown around which the clasp goes and upon that part of it that will be free from the antagonism of the opposite teeth. The side of the crown should be selected and marked by observations made in the mouth on the first visit. These can be fitted on the plaster cast.

When the impression in plaster has been taken of both clasps and plate, the easier plan will be to pour plaster and sand into it, and it is then exact, all ready for soldering.

Before any teeth are placed on it, by all means try it in the mouth to see if it will go in and out; for unless the impression has held all the pieces in exact apposition, the plate will not go in or be removed easily. A little filing may be needed to help in the adjusting. Frequently, where the tip rests on the grinding-surface of the crown, the latter has to be ground to let it rest firmly, which keeps the plate from anything more than resting in direct contact with the gum. This must be adjusted very accurately, and the plate will act as a saddle on the gum to prevent riding. This rest prevents any changes of position of the clasp on the tooth, and also any chafing on its surfaces. It is an absolute necessity. It is better it should be soldered to the plate than upon the clasp, as there will be more steadiness; but it must not interfere with the spring of the clasp. The drawings will show the best place for them on the tooth-crown. They should be very strong, as the whole force of mastication falls upon them. Use eighteen-karat solder for every attachment. These tips can rest on either a gold or amalgam filling, or the body of the tooth. If the latter, the enamel may be cut to prevent the antagonizing tooth from touching the tip.

Where there is decay upon the tooth to be clasped, I prefer to use amalgam containing much gold in it. There need be no fear of galvanic action or shock so long as the clasp is in direct contact with the amalgam.

My long experience with amalgam in these cases assures me that there is no action between these widely dissimilar metals to deteriorate their qualities as preservers of tooth-substance, but the reverse; and the gold amalgam does not discolor to any extent.

I prefer to allow the edge of the filling to stand outside of the clasp and not rest underneath it at the top or next the grinding-surface, and I do not hesitate to use the corundum wheel upon the enamel where slight projections interfere with a clasp resting securely. No harm can result where the cut surface is polished. If caries should occur at any point thereafter from accumulation of food, I should fill with amalgam. But this need not often result when cleansed after each meal.

As I have already stated, the injury done to the tooth where a clasp is upon it is from the food being allowed to remain for weeks in contact,—never from the clasp where it touches, unless too accurately fitted.

Each case must be thoroughly studied after the plaster cast is made, or the result will not be satisfactory. The points on the clasp and plate where the bar is soldered to connect them *are the vital parts*, and, unless judiciously chosen and the bar made of platinized gold wire and the base plate of two pieces of gold soldered together to stiffen it, and the clasp of proper width and thickness, the strain placed upon the mechanism will break it. The bar holding the clasp and plate must always be upon the side of the tooth where there will be least resistance. Take a second inferior molar that has tipped forward very much and also inclines to the tongue. Here the soldering should be done as far back on the buccal side of the clasp as can be accomplished. Then the spring of the clasp is not needed for the buccal side, but for the anterior and lingual sides, where projecting from a perpendicular. If soldered from the lingual side, it would be impossible to get the clasp on or off.

In the upper cases it is generally the reverse, although there are many exceptions, and no rigid rules can be laid down. Each one must be specially studied, or no good results. Nor can you rely upon fitting plate and clasp to the plaster-cast and soldering from that,—no, never do it! Take the trouble to take impression of both plate and clasp in the mouth, and then solder from that.

One of the greatest advantages, and one least likely to need repairing, is in the use of English crown-teeth used for rubber, or the tube-tooth for gold plate work. When vulcanized on, or soldered with backing, the grinding-surfaces are of porcelain, and are more artistic and sightly. Besides, if needing repair, it can readily be done. But when care is taken to make the plate heavy, and a stiff bar is used to connect plate and clasp, repairs are seldom needed. I prefer the English tooth, where no soldering is needed to attach it to the plate.

Above all else, the operator is clear from such vandalism as is practised for permanent bridge-work, and has infinitely more pride in the result. Spaces can be filled with satisfaction to patients and for far less money, and the profits be none the less.

A study of the cuts will give an idea of this work, but it will not appear so clear until it is attempted. The articulation for one or two teeth I do directly in the mouth, but for three or more I prefer my articulator, and put on the minute details after the teeth have been attached.

The letters on each cut have reference to the same parts on all. Fig. 1 is a cast for first upper bicuspid, right side. A filling of gold was placed in the distal surface of the natural cuspid with a hole, *c*, drilled into it for the pin *c* in Fig. 2. The second bicuspid had

Fig. 1.

Fig. 2.

also a large amalgam filling, around which the clasp was placed, so that it would not show from the mouth. Fig. 2 gives the plate with English crown thereon, with pin soldered to the plate. The clasp has a tip at *h* soldered to it, and *i* is the heavy platinized gold bar, showing how it forms the attachment between plate and clasp, and just where; *c* is a pin, soldered directly to the plate, which enters the hole in the gold filling shown in Fig. 1.

Where no filling is in the cuspid I should use a short clasp fitted near the cervix, to reach from the palatal surface to the buccal, where it would not show from the outside, and soldered on the extreme palatal side to gain a spring.

Fig. 3 is the skeleton plate without the crown, which shows clearly the cast for which it was made. (See Fig. 5.) In Fig. 3 is *e*, the tip, resting on the second molar, soldered to the plate. On the plate next to the second bicuspid is soldered an upright with a tip, *e*, and a thin, narrow projection underneath it, which sets in a groove shown at *d* in Fig. 5, in an amalgam filling, to keep the anterior of plate in position and to prevent the plate from pressing too hard upon the gum; *i* is the bar connecting plate and clasp on the lingual side. One or more pins for the crown can be used.

Fig. 3.

Fig. 4.

Fig. 4 shows the same with the crown cemented on with oxyphosphate, or vulcanized, or with gutta-percha.

Fig. 5 is a case, left side, lower jaw.

Fig. 5.

Fig. 6.

Fig. 6 is a second bicuspid tooth, right side, lower jaw. The bar *i* is soldered to the plate and clasp on the buccal side and the tip on the clasp on the first molar, and, as the crown is made entirely of gold, the tip is soldered directly to it to rest on the first bicuspid, and the anterior surface of the gold crown is made concave to fit into the distal surface of the first bicuspid, which prevents any movement laterally. A gold crown is used, as it is not seen, and facilitates greatly the soldering and adds immensely to the strength, and there is no danger of repairing in the future. The back tip, which rests on the molar, should have been soldered to the crown also, and less strain would come on the clasp.

Fig. 7 is an extreme case of tipping of the third molar, lower jaw, right side. The clasp was soldered to the plate on the buccal surface, and the plate at the second bicuspid was held as in Fig. 4. It could have been done by a narrow clasp to reach only partially around the second bicuspid, where it would not show on buccal side.

Fig. 8 is another extreme case where the second molar in the lower jaw projects towards the tongue and the second bicuspid towards the cheek. In this case the bar should be soldered on the buccal of the molar near its distal proximal surface at *b*, and the second bicuspid on the lingual surface at *a*.

Fig. 7.

Fig. 8.

I cannot urge too strongly the retention of all roots that can be made healthy when no crowns can possibly be placed on them. When allowed to remain, and these plates fitted directly upon them, they become firm and non-irritant, and enable the same pressure to be used on the artificial teeth as on the natural ones, and are clean as any part of the mouth. I seldom remove a root that can be reclaimed. The satisfaction to the patient is immense. The retention of one tooth, either with natural crown or artificial, is enough to hold in position a full upper set with a plate very narrow and confined alone to the alveolar border, and with no suction, provided the articulation is perfect.

I can further assure the far more perfect success of these operations if the clasps are made to touch not more than at three or four points on the crown. Where fitted accurately, caries is doubly invited by capillary action.

Not least of all the virtues of this class of operations is that the average dentist in plate-work, or even the operator, can learn to successfully do it, when but few can pretend to do a respectable piece of bridging.

While it is so easily done if nice care be used, I do not wish to be understood that I would have any tooth extracted, knowing that it could be replaced so handily and to such perfect satisfaction to the patient.

I wish it further understood that the patients for whom I did these operations were not mine originally. I am thankful that from the very first of my career I have held the human tooth so sacred, and as years advance I am jealous of every root that can be at all utilized; and I believe I am clean in such matters, and keep my patients so, or I could not have laid claim to have extracted so few teeth from any cause. Whatever there may be about this method that is original, I freely tender it to the profession as not only worthy their serious attention, but free from any incumbrance.

## Figures and Tables

**Fig. 1. f1:**
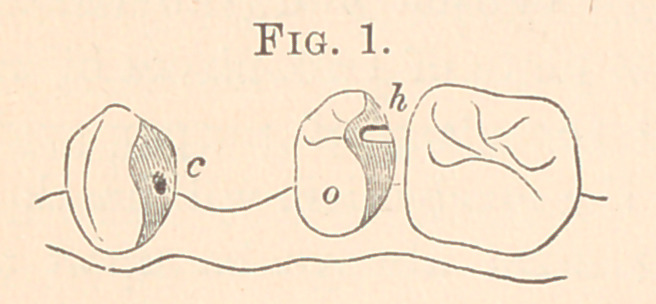


**Fig. 2. f2:**
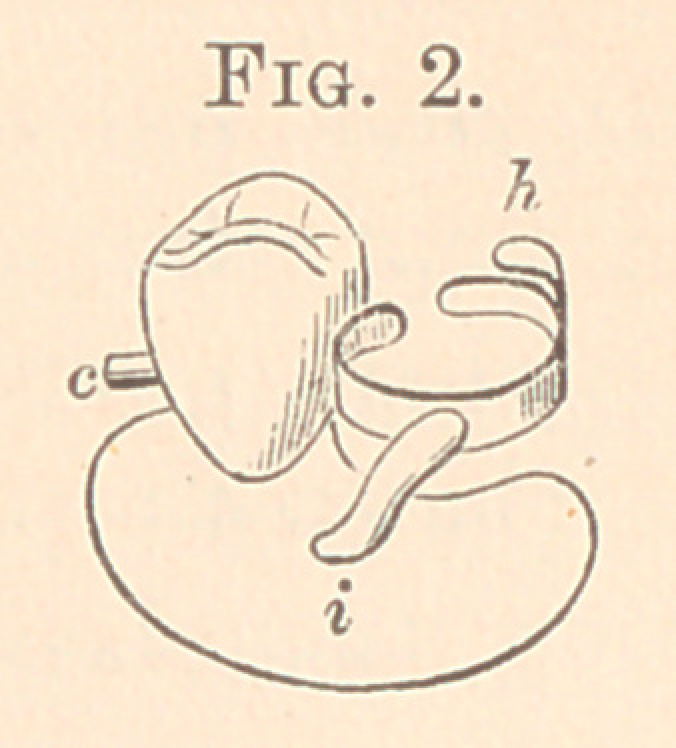


**Fig. 3. f3:**
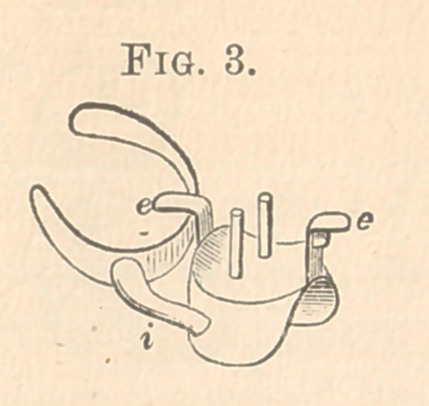


**Fig. 4. f4:**
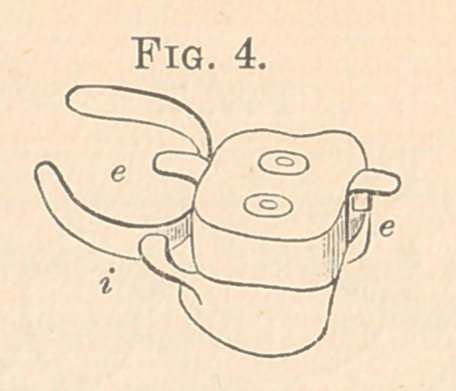


**Fig. 5. f5:**
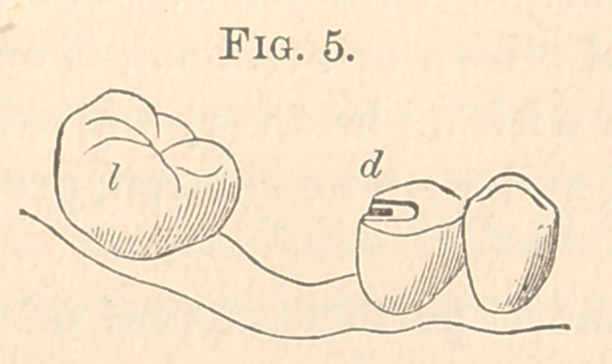


**Fig. 6. f6:**
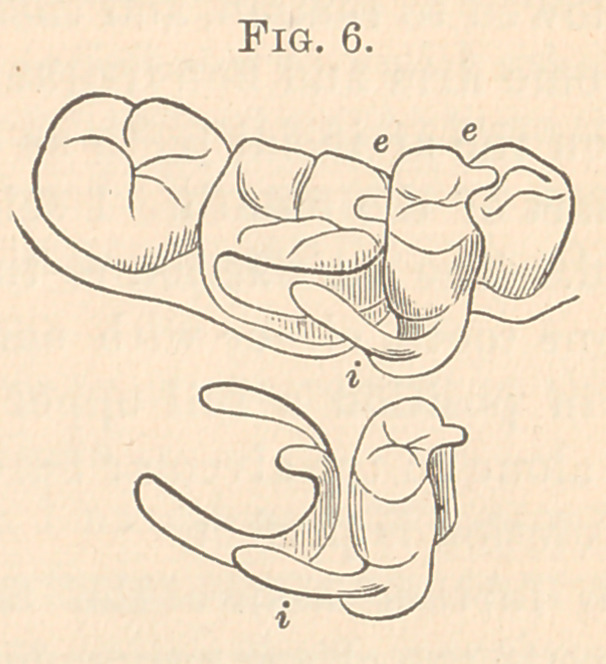


**Fig. 7. f7:**
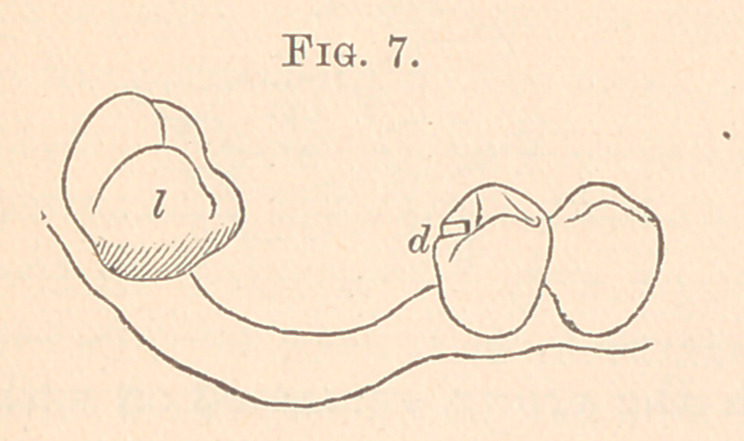


**Fig. 8. f8:**